# Effective Responder Communication Improves Efficiency and Psychological Outcomes in a Mass Decontamination Field Experiment: Implications for Public Behaviour in the Event of a Chemical Incident

**DOI:** 10.1371/journal.pone.0089846

**Published:** 2014-03-04

**Authors:** Holly Carter, John Drury, Richard Amlôt, G. James Rubin, Richard Williams

**Affiliations:** 1 Emergency Response Department, Public Health England, Porton Down, Salisbury, United Kingdom; 2 School of Psychology, University of Sussex, Falmer, Brighton, United Kingdom; 3 King's College London, Department of Psychological Medicine, London, United Kingdom; 4 Welsh Institute for Health and Social Care, University of South Wales, Pontypridd, United Kingdom; 5 Humanitarian and Conflict Response Institute, University of Manchester, Manchester, United Kingdom; 6 Extreme Events and Health Protection Section, Public Health England, London, United Kingdom; University of St Andrews, United Kingdom

## Abstract

The risk of incidents involving mass decontamination in response to a chemical, biological, radiological, or nuclear release has increased in recent years, due to technological advances, and the willingness of terrorists to use unconventional weapons. Planning for such incidents has focused on the technical issues involved, rather than on psychosocial concerns. This paper presents a novel experimental study, examining the effect of three different responder communication strategies on public experiences and behaviour during a mass decontamination field experiment. Specifically, the research examined the impact of social identity processes on the relationship between effective responder communication, and relevant outcome variables (e.g. public compliance, public anxiety, and co-operative public behaviour). All participants (*n* = 111) were asked to visualise that they had been involved in an incident involving mass decontamination, before undergoing the decontamination process, and receiving one of three different communication strategies: 1) ‘Theory-based communication’: Health-focused explanations about decontamination, and sufficient practical information; 2) ‘Standard practice communication’: No health-focused explanations about decontamination, sufficient practical information; 3) ‘Brief communication’: No health-focused explanations about decontamination, insufficient practical information. Four types of data were collected: timings of the decontamination process; observational data; and quantitative and qualitative self-report data. The communication strategy which resulted in the most efficient progression of participants through the decontamination process, as well as the fewest observations of non-compliance and confusion, was that which included both health-focused explanations about decontamination and sufficient practical information. Further, this strategy resulted in increased perceptions of responder legitimacy and increased identification with responders, which in turn resulted in higher levels of expected compliance during a real incident, and increased willingness to help other members of the public. This study shows that an understanding of the social identity approach facilitates the development of effective responder communication strategies for incidents involving mass decontamination.

## Introduction

The likelihood of incidents involving chemical, biological, radiological, and nuclear (CBRN) agents has increased in recent years, due to advances in technology [1], and the willingness of terrorists to obtain and use CBRN materials [2], [3]. Interventions designed to reduce the risk from CBRN agents, such as decontamination, may be more stressful for those involved than the incident itself, if not managed appropriately [4]. This may result in increased anxiety and reduced compliance during incidents involving decontamination [5], [6], [7], which could have serious consequences during an incident involving mass decontamination; failure of members of the public to behave cooperatively during mass decontamination may result in disorder, increased spread of any contaminant [8], and potentially increased numbers of dead and injured.

Despite this, planning for incidents involving mass decontamination has focused almost exclusively on technical aspects of decontamination, with little attempt to understand public feelings and behaviour [9]. The aim of the study described in this paper was to employ an experimental design to test the effect of three different responder communication strategies on public experiences and behaviour during a mass decontamination field experiment.

Decontamination involves those who have potentially been contaminated being asked to undergo a shower, in order to remove any contaminant from the skin. This reduces the risk of the agent being absorbed into the skin and causing further harm, and also reduces the risk of secondary contamination of other people and places. In the UK, the Fire and Rescue Service (FRS) have specially designed mass decontamination (MD1) units, which can facilitate the decontamination of up to 150 people per hour [10].

Findings from small-scale incidents involving decontamination have shown that failure of emergency responders to communicate effectively with members of the public, and to respect public concerns about privacy, can result in non-compliance and increased anxiety among members of the public [5]–[7]. Incidents involving mass decontamination may present further challenges for emergency responders since they involve crowds [8], which may be perceived by responders as a source of disorder and ‘panic’ during emergencies [11].

While the concern about disorder and panic is in line with early theories of crowd behaviour [12], [13], contemporary theories instead suggest that behaviour in emergencies is usually normatively structured [14]–[17], and shaped by group identities [18]. Specifically, self-categorization theory [19] suggests that, under certain conditions, those involved in such disasters perceive that they have a common fate, around which they categorize themselves as members of the same group (those affected by the disaster). The social identity model of collective resilience [18], [20] has been applied to various mass emergencies; results from this research show that shared identity, based on a sense of common fate, can result in increased helping and cooperative behaviour among those affected [18], [20].

In the study described in this paper, we apply the social identity approach to mass decontamination. This approach not only facilitates an understanding of how shared identity among group members can enable the development of shared group norms, but also of how interactions between different groups can shape the norms, and hence behaviour, of a group. This is likely to be particularly relevant to incidents involving mass decontamination, since mass decontamination is an intergroup situation (members of the public vs. emergency responders), in which one group (emergency responders) are trying to direct the behaviour of another (members of the public) [21].

Research in related domains of crowd behaviour suggests that communication is a key intervention through which emergency responders can improve the management of the decontamination process, as effective communication results in increased perceptions of responder legitimacy [22]. Applying the elaborated social identity model (ESIM) [23] to understand interactions between police and football supporters has shown that increased perceptions of police legitimacy result in increased identification with police [24], which in turn results in increased compliance with police instructions [25].

While shared identity is likely to be present among members of the public during disasters, as a result of the sense of shared fate they all face [18], [20], research has also shown that increased identification with emergency responders can result in increased identification with other members of the public, possibly because members of the public may unite around their shared identification with emergency responders [21]. Shared identity among members of the public around a shared identity with emergency responders is likely to play a key role during incidents involving mass decontamination. This is because if members of the public unite around their shared identity with emergency responders, they will internalise the aims of responders (e.g. to facilitate effective decontamination of all those potentially contaminated), which will become shared goals of the group; the internalisation of decontamination as a shared group goal will result in increased cooperative and helping behaviour during incidents involving mass decontamination.

There are two other ways in which shared social identity may be of benefit during incidents involving mass decontamination. First, shared identity may facilitate a sense of collective agency among members of the public [18], [26], enabling members of the public to work together to achieve the shared goal of decontamination, and thus increasing compliance [27]. This will be crucial during a real life incident involving mass decontamination, in which emergency responders will have insufficient resources to force members of the public to undergo decontamination; a belief that the group can work together to achieve shared norms and goals (e.g. decontamination) will therefore promote willingness to comply with responder instructions, and hence facilitate orderly and efficient decontamination. Second, shared identity may reduce public anxiety by increasing shared expectations of support [26], [28], [29], and enabling members of the public to work together to challenge and reduce shared stressors [30]. Reduced anxiety about decontamination may also increase compliance with the process [27].

### The present study

As noted above, decontamination has traditionally been seen as a technical issue [9]. Planning for such incidents has been based on assumptions about likely crowd behaviour (e.g. ‘mass panic’), which has resulted in a focus on controlling, rather than communicating with, members of the public [9]. There is therefore a need for research to examine the effectiveness of different communication strategies, to ensure that plans for the management of decontamination are based on evidence, rather than assumptions. Social psychological theories, in particular the social identity approach, provide a useful basis for understanding how different responder communication strategies might affect public experiences and behaviour during incidents involving mass decontamination, and hence affect the successful management of such incidents.

Previous research that has attempted to examine hypotheses relating to the effect of social identity processes during mass decontamination has involved purely self-report, rather than behavioural, measures [21]. Further, previous research which has used an experimental design to test the effect of different communication strategies has employed an online visualisation design [31], and may therefore have lacked ecological validity.

The present study extended previous research [21] by asking participants to actually undergo a decontamination shower during a mass decontamination field experiment, in which the effect of three different responder communication strategies were tested. To increase realism, participants were decontaminated within an MD1 showering unit, such as would be used by the Fire and Rescue Service (FRS) during a real life incident involving mass decontamination, and the decontamination process was managed by members of the East Sussex FRS, who were dressed in Personal Protective Equipment (PPE). Asking participants to actually undergo a decontamination shower not only increased the ecological validity of the research, but also enabled behavioural measures, such as observations of participant behaviour, and measures of the speed and efficiency of the decontamination process, to be collected alongside self-report measures. Thus the present study combines an experimental research design with a realistic scenario in order to examine both participant experiences and behaviour during mass decontamination.

During the field experiment, the effect of three different responder communication strategies on public experiences and behaviour during the decontamination process was tested. The ‘theory-based’ communication strategy used in this research was designed based on the recommendations derived from the literature [27], [31], and included health-focused explanation about why the decontamination process was necessary, regular updates on the actions emergency responders were taking, and sufficient practical information. The ‘standard practice’ communication condition was based on current practices, and included sufficient practical information, but no health-focused explanation or information about actions emergency responders were taking. The ‘brief’ communication condition was designed to reflect a ‘worst case’ communication strategy (as has been observed during field exercises involving mass decontamination), which included no health-focused explanation, no updates on actions emergency responders were taking, and only very basic practical information.

In line with previous research showing that increased information resulted in quicker, more efficient evacuations from a railway station during a simulated fire evacuation [32], in the present study, it was expected that the decontamination process would progress most efficiently in the theory-based communication condition. The optimum time for members of the public to undergo the decontamination process is 10 minutes. However, the optimum time in the current study was 9 minutes and 30 seconds, because the decontamination unit used in this study differed slightly from the standard FRS decontamination unit in terms of the time which participants spent in each section. A quicker time might mean that decontamination has not been carried out effectively, while a slower time could result in delays to the process, and could cost lives. It was therefore expected that those in the theory-based communication condition would progress through the decontamination process in a time closest to 9 minutes 30 seconds.

In line with findings from small scale incidents involving decontamination [5]–[7], it was expected that the observational data would show greater compliance and less confusion among participants in the theory-based communication condition. Further, in line with the principles of the social identity approach [18], [20], it was expected that participants would be more willing to help each other in the theory-based communication condition, and therefore that more helping behaviours would be observed among participants. However, it was also thought possible that there might be less helping behaviours observed among participants during the theory-based communication condition, since participants would receive more information and communication from responders in this condition, thereby reducing the need for participants to help each other.

In terms of the self-report measures, it was expected that those in the theory-based communication group would report more positive outcomes (e.g. increased: responder legitimacy; identification with emergency responders; identification with other members of the public; expectations of collective agency; expectations of compliance; willingness to help others; and decreased actual anxiety experienced, and expectations of anxiety during a real incident), than those in the standard practice or brief communication groups. In turn, it was expected that those in the standard practice communication group would report more positive outcomes than those in the brief communication group.

It was also expected that those in the theory-based communication group would report more positive outcomes at time 2 (post-communication intervention) than at time 1 (pre-communication intervention), while those in the brief communication group would report more negative outcomes at time 2 than at time 1. As the standard practice condition was designed to reflect current standard practice, it was expected that those in the standard practice communication group might report similar outcomes at time 2 as at time 1.

**Figure 1:**
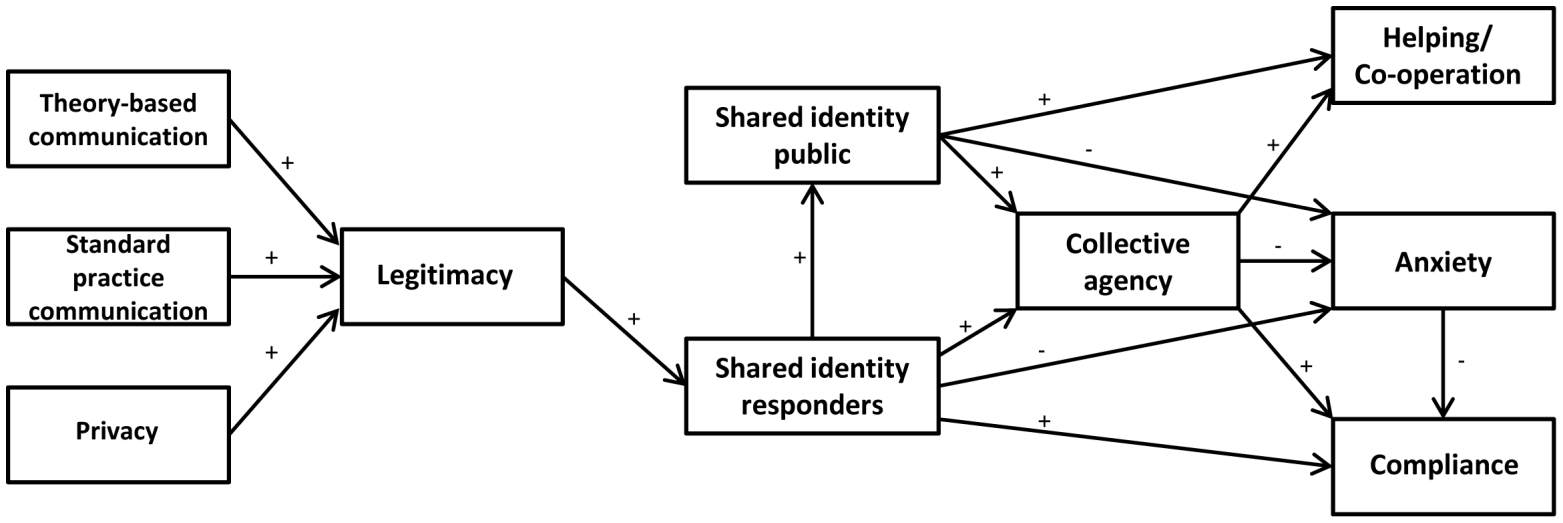
A path model representing the expected relationships between variables.

A path model was created to illustrate the predicted relationships between variables, based on the previous literature, and this is presented in Figure 1. The ‘theory-based communication’ variable is in comparison to the standard practice and brief communication conditions, while the ‘standard practice communication’ variable is in comparison to the brief communication condition. Plus and minus signs indicate the direction of the expected relationship between variables. The model shows expected relationships between (i) theory-based communication (compared to standard practice and brief communication) and legitimacy, and (ii) between standard practice communication (compared to brief communication) and legitimacy. This is based on previous findings using the ESIM, as described above, which can be applied to suggest that increased communication from emergency responders will promote a perception that responders are behaving legitimately. The model also shows an expected relationship between perceptions of privacy and legitimacy, since it is expected that having sufficient privacy will result in a perception that participants are being treated fairly by emergency responders. The other expected relationships in the model are based on the social identity approach (as described above), and suggest that perceptions of responder legitimacy will result in identification with emergency responders, which will in turn result in identification with other members of the public, and collective agency. It is expected that collective agency will result in increased public compliance, increased helping and cooperative behaviour, and reduced public anxiety.

## Methods

### Ethics statement

Ethical approval was obtained from the University of Sussex Psychology and Life Science Ethics Committee. Before taking part in the study, participants were asked to read an information sheet about the research, and complete a consent form to indicate their informed consent to participate. Participants were informed that any information they provided would be confidential, and that they were free to withdraw from the study at any time.

### Design

A mixed between- and within-subjects design was used. The between-subjects experimental design had one factor (quality of communication), with three levels (theory-based, standard practice and brief). The within-subjects design had one factor (time), with two levels (time 1, before undergoing decontamination; time 2, after undergoing decontamination). Four different types of data were collected for participants in each group: timings for how long each participant took to progress through the decontamination process; observational data relating to incidences of non-compliance, confusion, and helping behaviours; quantitative questionnaire data; and qualitative questionnaire data. For the quantitative self-report data, the dependent variables were: perceptions of responder legitimacy, identification with emergency responders, identification with other members of the public, perceptions of collective agency, willingness to help others during a real incident, expectations of compliance during a real incident, and expectations of anxiety during a real incident.

### Participants

A self-selected sample of 111 students from the University of Sussex were recruited using the University of Sussex online system for recruiting research participants, email, Twitter, Facebook, and recruitment posters. Participants signed up to one of three different timeslots, without knowing which timeslot corresponded to which communication condition, or even knowing that there were three different communication conditions (theory-based: n = 42, standard practice: n = 32, brief: n = 37). Participants received course credit or a £20 high street gift voucher for taking part in the research.

### Materials

#### Scenario

A scenario involving a potential chemical release within a University lecture theatre was developed by the research team, and was then discussed with a senior Exercise Planner, who has extensive experience of writing scenarios for emergency preparedness exercises. The scenario was then pilot tested using an online visualisation study, and was perceived to be highly realistic [27], [31]. The scenario contained a description of the initial phase of the incident, up to and including FRS responders setting up a decontamination tent. This was designed to set the scene for participants, and to allow them to visualise that they had been involved in the type of incident described. See Appendix S1 for a copy of the scenario used during this study.

#### Communication intervention

Three different communication interventions were developed, which were designed to reflect different communication strategies which responders might use during a real incident involving mass decontamination. These were then pilot-tested using an online visualisation experiment, to ensure that each condition was perceived as intended [27], [31]. See Appendix S2 for a copy of the three communication interventions used during this study.

#### Pre-communication intervention questionnaire

The pre-communication intervention questionnaire contained items relating to: perceptions of responder legitimacy (e.g. “ I think that the emergency responders will treat people with respect during the decontamination process today”) (4 items, α = .83); identification with emergency responders (e.g. “I feel a sense of unity with the emergency responders who will be managing the decontamination process today”) (3 items, α = .83); identification with other members of the public (e.g. “I identify with the other volunteers who are taking part in the trial today”) (3 items, α = .75); and expectations of anxiety during a real incident (e.g. “If a real incident of this kind were to occur, I would feel nervous”) (3 items, α = .79).

#### Post-communication intervention questionnaire (quantitative items)

The post-communication intervention questionnaire contained items relating to: perceptions of responder communication (manipulation check) (e.g. “The emergency responders explained clearly what was happening during the decontamination process”) (2 items, *r* = .87); perceptions of communication messages (manipulation check) (e.g. “I understood why I was being asked to go through the decontamination process”) (2 items, *r* = .85); sufficiency of practical information (manipulation check) (e.g. “I was provided with sufficient practical information about what I was supposed to do during the decontamination process”) (3 items, α = .94); engagement with the study (manipulation check) (“I felt emotionally engaged during the trial”) (1 item); perceptions of privacy (“I had sufficient privacy during the decontamination process”) (1 item); perceptions of responder legitimacy (e.g. “I felt that the emergency responders behaved in a fair way towards us during the decontamination process”) (4 items, α = .91); identification with emergency responders (e.g. “I felt that I had a lot in common with the emergency responders who were managing the decontamination process today”) (3 items, α = .93); identification with other members of the public in the trial (e.g. “I felt a sense of unity with the other volunteers who took part in the trial today”) (3 items, α = .89); collective agency (e.g. “ I felt able to work with other volunteers to successfully undergo the decontamination process”) (2 items, *r* = .95); willingness to help others during a real incident (“If this was a real incident, I would be willing to help other members of the public”) (1 item); expectations of compliance during a real incident (e.g. “ I would be willing to undergo a decontamination shower during a real life incident of this kind”) (3 items, α = .79); expectations of anxiety during a real incident (e.g. “If this had been a real incident, I would have felt worried”) (3 items, α = .87); and actual anxiety experienced during the study (e.g. “I felt nervous during the decontamination process”) (3 items, α = .78).

#### Post-communication intervention questionnaire (qualitative items)

There were six qualitative items on the post-communication intervention questionnaire. These were: “Please explain any ways in which you feel communication/information during the decontamination process could have been improved”; “If you would not be willing to undergo a decontamination shower during a real incident, or would not be willing to be naked inside the decontamination showers in a real incident, please explain why”; “Please describe any ways in which emergency responders could have improved the way they dealt with the decontamination process”; “Please explain any instances when you saw volunteers co-operating. Include any instances when you gave help to another volunteer, or received help from another volunteer”; “If you felt nervous or worried, please describe what the main reason for this was”; and “Please describe any ways in which you feel this trial could have been improved.”

### Procedure

Before taking part in the experiment, participants received briefing instructions, informing them about the nature of the research, and that they would be required to undergo a decontamination shower. The experiment took place in a vacant car park on the University of Sussex campus, where an MD1 decontamination unit had been set up by members of the research team. Nine members of the East Sussex FRS agreed to assist with the experiment, in order to increase the realism of the scenario.

Participants took part in the experiment in one of three different timeslots, each corresponding to a different communication condition (theory-based, standard practice, or brief). The brief condition took place first, followed by the standard practice condition, and then the theory-based condition. Although responders were briefed on how to act during each condition, it is possible that the way in which they managed the incident might have improved slightly through practice over the three conditions. Participants in each group received a briefing presentation, in which they listened to the scenario, were shown a picture of members of the public going through an MD1 decontamination tent, and were asked to visualise that they had been involved in the incident described. Participants then completed the pre-communication intervention questionnaire, before listening to the scenario a second time. Following this, three responders from East Sussex FRS, dressed in full personal protective equipment (PPE), entered the briefing room to escort participants outside, to where the decontamination process would take place. Participants then experienced a 20 minute pre-planned ‘delay’, during which time participants were asked to disrobe. In the present study, participants were asked to disrobe down to their swimwear, to protect their modesty; in a real incident, those affected would be asked to fully disrobe, and would be naked during the decontamination process. Participants received one of three different communication interventions. Those in the theory-based communication condition received regular messages over loudspeaker, including health-focused explanations about why the decontamination process was necessary, and the actions emergency responders were taking. The 20 minute pre-planned delay was designed to represent the quickest time in which FRS responders could realistically arrive at the scene and begin to manage the incident during a real life situation; this was introduced in order to increase the realism of the scenario.

Participants in each condition entered the decontamination shower in groups of ten, until all participants in that condition had been decontaminated. The last participants in each condition went through the shower in smaller groups, as the participant numbers within each condition were not evenly divisible by ten (e.g. if there were 38 participants in a condition, the first three groups went through the shower as a group of ten, and the last group went through the shower as a group of 8). On beginning the decontamination process, participants in the theory-based communication condition received practical information messages, via loudspeaker, which included full details of the actions they were expected to take during the decontamination process. In contrast, those in the standard practice communication condition received only two update messages during the initial 20 minute wait (one at the beginning of the delay, and one at the end), and the same practical information messages as those in the theory-based communication condition, while those in the brief communication condition received the same irregular updates during the initial 20 minute waiting period as those in the standard practice condition, and only basic practical information during the decontamination process. As well as receiving the different messages over loudspeaker, participants in each of the three different groups also received different treatment from the FRS responders. Prior to the study, FRS responders were briefed to be as helpful and communicative as possible in the theory-based communication condition, provide only practical information and no extra communication during the standard practice condition, and provide no extra information or help in the brief condition.

Video footage was collected during the initial 20 minute waiting period, and during the decontamination process itself, to allow observational analysis to be conducted. Six different video cameras were used: two cameras were positioned at the disrobe end of the MD1 decontamination tent; two cameras were positioned within the two showering sections of the decontamination tent; and two cameras were positioned at the rerobe end of the decontamination tent. Each group of participants (10 per group) was also timed going through the decontamination process, by a member of the research team, who recorded the time each group entered and exited the decontamination unit, using a stopwatch. Participants were timed from the point they entered the disrobe section of the decontamination tent, to the point they exited the rerobe section of the decontamination tent.

Following the decontamination process, participants were escorted to a changing area, and were then asked to complete the post-communication intervention questionnaire. Participants then received a debriefing statement about the research.

### Analysis

The quantitative questionnaire data, including the pre- and post-communication intervention data and the manipulation checks, and the experiment timing data were analysed using SPSS 20. The quantitative questionnaire data were also analysed using AMOS 19, which was used to create a path model.

The qualitative questionnaire data were analysed using content analysis. Based on the hypotheses of the study, four relevant coding categories were identified: did participants say they received sufficient overall communication from emergency responders; did participants say they received sufficient practical information from emergency responders; did a lack of communication contribute to any anxiety experienced by participants; and did participants feel confused during the process. See Appendix S3 for some examples of the types of material which were coded into each category.

A similar method was used to analyse the video observational data. Three behaviours of interest were identified prior to the study, based on the hypotheses: non-compliant behaviours (such as disobeying responder instructions), helping behaviours (such as providing another volunteer with information, or helping them to disrobe); and confusion (evidenced by hesitating prior to carrying out responder instructions, or asking another volunteer for clarification). Data were coded to show how many times behaviours of each type were observed during each of the three different communication conditions. See Appendix S4 for some examples of the types of material which were coded into each category.

All data presented in this paper are freely available upon request.

## Results

### Experiment timing data

The times taken for each group to complete the shower process were recorded, and a comparison was made between groups in each of the three communication conditions. As the timing data involved data collected from small groups of participants, rather than individuals, the data were not independent, and it was therefore not possible to carry out statistical tests of significance. We therefore examined the mean, minimum, maximum, and range data for group timings across the three conditions. The mean time taken for groups to progress through the showers in each condition was compared to the optimum time (9 minutes and 30 seconds).

Results revealed that the mean time for those in the theory-based communication condition was 1 minute 18 seconds longer than the optimum time, the mean time for those in the brief communication condition was 2 minutes 20 seconds longer than the optimum time, and the mean time for those in the standard practice condition was 5 minutes 20 seconds longer than the optimum time. Further, those in the theory-based communication group progressed through the process more consistently, with the slowest group in that condition taking only 1 minute 30 seconds longer than the quickest group. In contrast, the slowest group in the standard practice condition took 6 minutes 30 seconds longer than the quickest group, while the slowest group in the brief condition took 11 minutes 18 seconds longer than the quickest group. This is partly due to the fact that the quickest group in the brief condition took only 8 minutes 30 seconds to complete the process, which was 1 minute quicker than the optimum time, and raises questions as to whether the process was completed appropriately. Thus, as predicted, the theory-based communication strategy resulted in the quickest and most efficient progression of volunteers through the decontamination process. See Table S1 for the mean, standard deviation, min, max, and range times for the three different communication conditions. See Appendix S5 for the timing data for each of the groups within each condition.

### Observational data

Two observers analysed the data, and a test of inter-rater reliability revealed that there was a 74% agreement rate between the two observers. A chi-squared test revealed that the difference between the scores of the two raters was not significant (*χ^2^*(1df) = .12). As expected, non-compliant behaviours (e.g. disobeying responder instructions) were observed more often in the brief communication condition than in either the standard practice or theory-based communication conditions (see Table S2). The difference between groups was significant (*χ^2^*(2df) = 22.36, *p*<.001). Post-hoc tests revealed that this was due to a significantly higher number of non-compliant behaviours in the brief condition compared to both the standard practice condition (*χ^2^*(1df) = 9.85, *p*<.05) and the theory-based condition (*χ^2^*(1df) = 15.70, *p*<.001). Similarly, behaviours indicative of confusion (e.g. asking others what to do before carrying out actions) were also observed most often in the brief communication condition, and were observed more commonly in the standard practice communication condition than in the theory-based communication condition. The difference between groups was significant (*χ^2^*(2df) = 13.32, *p*<.05). Post-hoc tests revealed that this was due to a significantly higher number of incidences of confusion in the brief communication condition compared to both the standard practice condition (*χ^2^*(1df) = 4.40, *p*<.05) and the theory-based condition (*χ^2^*(1df) = 12.63, *p*<.001). Observed helping behaviours were also higher in the brief communication group than in the other two groups, although the difference between groups was not significant (*χ^2^*(2df) = 3.06). Possible reasons for this are outlined in the discussion.

### Quantitative questionnaire data

#### Manipulation checks

Participants in all groups reported good engagement with the study, with a mean scale score for engagement of 4.59 which was significantly higher than the mid-point value of 4, *t*(110) = 4.33, *p*<.001. There were no significant differences in engagement between the three groups.

MANOVA indicated that there were no significant differences between the three communication groups on any of the variables which were measured at time 1 (prior to receiving the communication intervention during decontamination) (shared identity with members of the public; shared identity with responders; legitimacy; and anxiety). There were also no significant differences in perceptions of privacy between the three different communication groups, *F*(2, 108) = 1.11.

To check whether the manipulations of communication were perceived in the ways intended, MANOVA was carried out on perceptions of communication with responders, communication messages (provided over loudspeaker), and practical information. This revealed that there were some significant differences between groups, *F*(6, 214) = 8.56, *p*<.001. When the results for the three dependent variables were considered separately, it was revealed that there were significant differences in perceptions of communication from responders between groups, *F*(2, 108) = 26.10, *p*<.001, with the theory-based communication group (*M* = 5.81) reporting significantly better perceptions of communication from responders than either the standard practice communication group (*M* = 3.38, *p*<.001) or the brief communication group (*M* = 3.80, *p*<.001). There were no significant differences in perceptions of communication from responders between the standard practice and brief communication groups. There were also significant differences in perceptions of communication messages between groups, *F*(2, 108) = 13.12, *p*<.001, with the theory-based communication group (*M* = 5.61) reporting significantly better perceptions of communication messages than either the standard practice communication group (*M* = 3.78, *p*<.001) or the brief communication group (*M* = 4.14, *p*<.001). There were no significant differences in perceptions of communication messages between the standard practice and brief communication groups. There were also significant differences in the perception of the provision of practical information between groups, *F*(2, 108) = 19.61, *p*<.001, with the theory-based communication group (*M* = 6.09) reporting significantly better perceptions of the provision of practical information than either the standard practice communication group (*M* = 4.29, *p*<.001) or the brief communication group (*M* = 4.14, *p*<.001). There were no significant differences in the perception of the provision of practical information between the standard practice and brief communication groups. The manipulation checks therefore showed that the theory-based communication message had been perceived as intended, but that participants had not perceived any differences between the standard practice and brief communication messages.

#### Between groups analysis

MANOVA was carried out to test for predicted differences between the three different communication groups on the variables measured at time 2 (after receiving the communication intervention during decontamination) (see Table S3 for the variable mean scores and standard deviations at time 2 for the three different communication conditions). This revealed that there were some significant differences between the three different communication groups, *F*(18, 190) = 2.91, *p*<.001. When the results for the dependent variables were considered separately, it was revealed that there were significant differences in perceptions of responder legitimacy between groups, *F*(2, 102) = 19.99, *p*<.001, with those in the theory-based communication group (*M* = 6.41) reporting significantly higher perceptions of responder legitimacy than those in either the standard practice (*M* = 5.01, *p*<.001) or brief communication groups (*M* = 4.85, *p*<.001). There were no significant differences in perceptions of responder legitimacy between the standard practice and brief communication groups. There were also significant differences in identification with emergency responders between groups, *F*(2, 102) = 9.85, *p*<.001, with those in the theory-based communication group (*M* = 4.12) reporting significantly higher identification with responders than those in either the standard practice (*M* = 3.17, *p*<.05) or brief communication groups (*M* = 2.83, *p*<.001). There were no significant differences in identification with emergency responders between the standard practice and brief communication groups. There was a significant difference in expectations of anxiety during a real incident between groups, *F*(2, 102) = 3.01, *p* = .05. This was due to reduced expectations of anxiety in the theory-based communication group (*M* = 5.11) compared to the standard practice (*M* = 5.72) and brief (*M* = 5.73) communication groups, although the difference between individual groups was not significant.

Although there were no other significant differences between groups, the theory-based communication condition did generate higher mean values for compliance, collective agency, and willingness to help others during a real incident (see Table S3).

Results of between-groups analysis were broadly as expected, in showing that those in the theory-based communication condition reported higher mean scores of almost all variables, compared to the other two groups. However, results were not as expected in relation to the brief and standard communication conditions, as there were no significant differences between these two conditions.

#### Time 1 to time 2 differences

Within subjects *t*-tests revealed that there were some significant differences in variable mean scores from time 1 to time 2. There was a significant increase in perceptions of responder legitimacy in the theory-based communication group from time 1 (*M* = 6.15) to time 2, *M* = 6.41, *t*(40) = −2.07, *p*  = .05, and a significant decrease in responder legitimacy in the standard practice communication group from time 1 (*M* = 6.16) to time 2, *M* = 5.01, *t*(31) = 4.71, *p*<.001, and in the brief communication group from time 1 (*M* = 5.73) to time 2, *M* = 4.85, *t*(32) = 3.66, *p*<.05. There was a significant decrease in expectations of anxiety in the theory-based communication group from time 1 (*M* = 5.91) to time 2, *M* = 5.11, *t*(40) = 4.52, *p*<.001, a decrease in expectations of anxiety in the standard practice communication group from time 1 (*M* = 6.15) to time 2 (*M* = 5.72), which was not significant, and no significant change in expectations of anxiety in the brief communication group from time 1 to time 2. There was a significant increase in identification with emergency responders in the theory-based communication group from time 1 (*M* = 3.35) to time 2, *M* = 4.12, *t*(40) = −5.70, *p*<.001, and a non-significant decrease in identification with emergency responders in the standard practice group from time 1 (*M* = 3.59) to time 2, *M* = 3.17, *t*(31) = 1.78, and in the brief group from time 1 (*M* = 3.31) to time 2, *M* = 2.83, *t*(36) = 1.5. There was a significant increase in identification with other members of the public in all groups from time 1 to time 2 (theory-based: time 1 *M* = 3.87, time 2 *M* = 4.98, *t*(40) = −7.67, *p*<.001; standard practice: time 1 *M* = 4.32, time 2 *M* = 5.10, *t*(31) = −4.44, *p*<.001; brief: time 1 *M* = 4.21, time 2 *M* = 5.27, *t*(36) = −6.40, *p*<.001).

Results from the within-subjects analysis were therefore broadly as expected, in showing a significant increase in positive outcomes (perceptions of responder legitimacy, identification with other members of the public, identification with emergency responders), and a reduction in anxiety, in the theory-based communication condition from time 1 to time 2. Results were also broadly as expected in showing that this increase in positive outcomes, and reduction in anxiety, did not occur in either of the other two conditions.

#### Path analysis

The time 2 measures were entered into a path model, and the hypothesised path model was tested. Model chi-square was used to evaluate the overall model-data fit. To be said to have a good fit, a model should have a chi-square of greater than .05. However, chi-square is sensitive to sample size, and therefore a significant chi-square result does not necessarily mean that a model should be rejected [33]. Other fit indices (e.g. CFI, RMSEA) should be examined along with the chi-square value, to assess the overall fit of the model. Orthogonal contrast coding was used to create two categorical variables (‘theory-based communication’ and ‘standard practice communication’) out of the three different communication groups. The ‘theory-based communication’ variable was coded to compare the theory-based communication condition to the standard practice and brief communication conditions (theory-based condition = 2, standard practice condition = −1, brief condition = −1), and the ‘standard practice communication’ variable was coded to compare the standard practice communication condition to the brief communication condition (theory-based condition = 0, standard practice condition = 1, brief condition = −1). The ‘theory-based communication’ variable compares the theory based communication condition to the standard practice and brief conditions, and therefore includes data from all participants. However, the ‘standard practice communication’ variable compares the standard practice communication condition to the brief communication condition, and therefore excludes data from those in the theory based communication condition, in order to enable this comparison. The model aims to show how these two different comparison conditions affect perceptions of responder legitimacy. Path weights from perceptions of legitimacy onwards (the rest of the model) are based on data from all participants and show various predicted relationships between variables.

When all relationships in the hypothesised model were entered into the path model, this proved to have poor fit with the data, *χ* ^2^(27df) = 59.54, *p*<.001, CFI = .79, RMSEA = .10. To improve the fit of the model, non-significant paths were removed from the model. The main change to the hypothesised model was that anxiety was removed from the model, since the expected relationships between anxiety and the other variables were not present; possible reasons for this are outlined in the discussion section of this paper. Following the removal of non-significant paths, two extra paths were added to the model, based on possible explanations derived from the relevant theory and research. The first was a direct path between legitimacy and collective agency. It was expected, based on existing literature, that perceptions of responder legitimacy might contribute to a belief in the effectiveness of the decontamination process itself, and that this could result in increased collective agency. The second was a path between the error variables of collective agency and willingness to help others during a real incident. This path was added as it is possible that there might be a factor which contributes to both collective agency and willingness to help others, thus the error variables would be correlated. Modification indices were not used to improve the fit of the model, as there were some missing data points, and so it was not possible to use modification indices to improve model fit.

**Figure 2:**
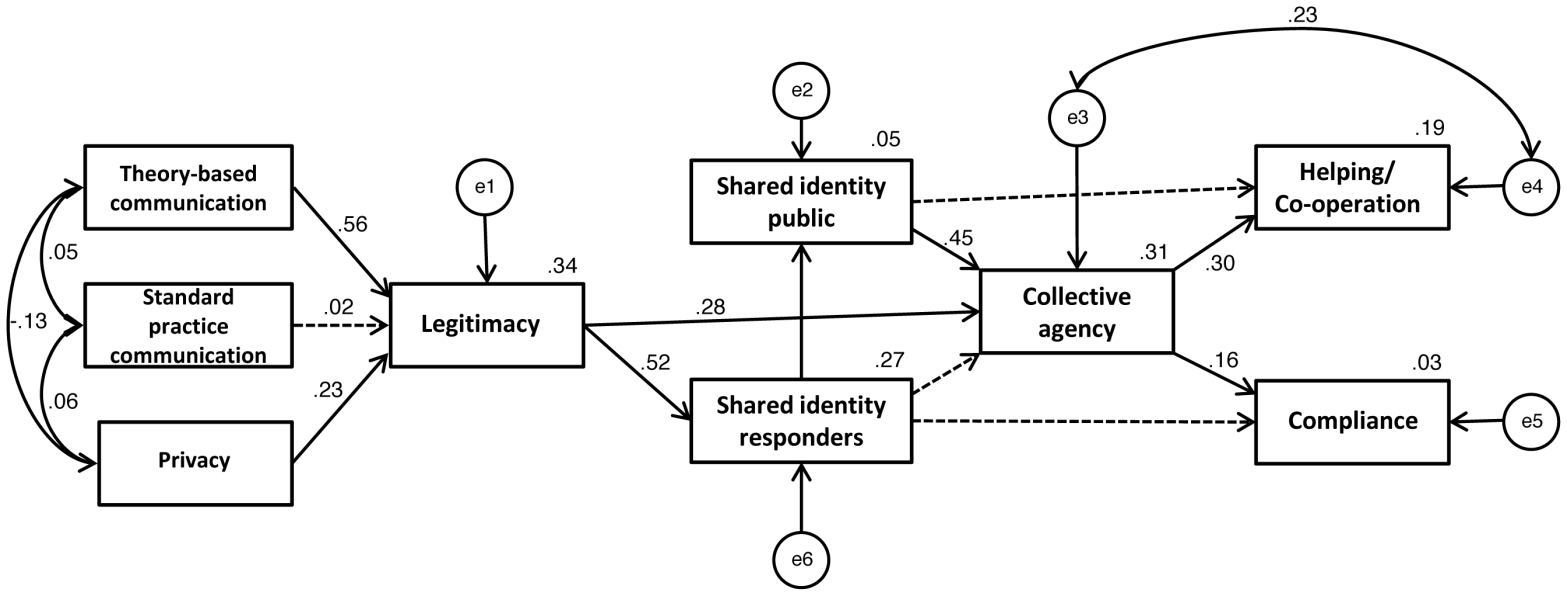
A path model of the data collected at time 2, following the mass decontamination field experiment.

The removal of non-significant paths and addition of two theoretically-justified paths improved the fit of the model. The improved model is presented in Figure 2.

The path model showed a reasonable overall fit with the data *χ^2^*(22) = 35.03, *p*<.05; CFI = .91; RMSEA = .07.

The model explains 34% of the variance in perceptions of responder legitimacy, 27% of the variance in identification with emergency responders, 5% of the variance in identification with other members of the public, 31% of the variance in collective agency, 3% of the variance in willingness to comply during a real incident, and 19% of the variance in willingness to help others during a real incident. There was no significant relationship between expectations of anxiety and any of the other variables, so expectations of anxiety during a real incident was not included in the model. As hypothesised, the model shows that being in the theory-based communication group, as opposed to the standard practice or brief communication groups, was a significant predictor of increased perceptions of responder legitimacy (*β* = .56, *p*<.001). However, being in the standard practice communication group, as opposed to the brief communication group, was not a significant predictor of perceptions of responder legitimacy (*β* = .02). As hypothesised, there was also a significant relationship between perceptions of sufficient privacy and perceptions of responder legitimacy (*β* = .23, *p*<.05), perceptions of responder legitimacy and identification with emergency responders (*β* = 0.52, *p*<.001), and identification with emergency responders and identification with other members of the public (*β* = .23, *p*<.05). The model also supported the hypotheses in showing a significant relationship between identification with other members of the public and collective agency (*β* = .45, *p*<.001) and between collective agency and willingness to help others (*β* = .30, *p* = .05). There was also a positive relationship between collective agency and compliance, although this was not significant (*β* = .16). There was also a significant direct relationship between perceptions of responder legitimacy and collective agency (*β* = .28, *p*<.001).

There was no significant direct relationship between identification with emergency responders and collective agency, identification with emergency responders and compliance, or identification with members of the public and willingness to help others. There were, however, indirect relationships between these variables, mediated by other variables within the model.

As predicted, social identity variables (perceptions of responder legitimacy, and collective agency) performed a significant mediating role within the model. Perceptions of responder legitimacy significantly mediated the relationship between being in the theory-based communication group and identification with emergency responders *b* = .33, BCa CI [.15, .52], *k^2^* = .21, 95% BCa CI [.10, .33], and being in the theory-based communication group and collective agency *b* = .17, BCa CI [.02, .35], *k^2^* = .14, 95% BCa CI [.02, .27]. Collective agency significantly mediated the relationship between identification with other members of the public and willingness to help others during a real incident *b* = .20, BCa CI [.10, .33], *k^2^* = .23, 95% BCa CI [.13, .40].

An alternative model was also tested, to rule out other possible explanations for the relationships between variables. It is possible that the relationship between perceptions of responder legitimacy and identification with responders might be the opposite way to that depicted in Figure 2; identification with emergency responders might predict perceptions of responder legitimacy, rather than perceptions of responder legitimacy predicting increased identification with emergency responders. We therefore tested an alternative model, in which the relationship between communication condition, perceptions of legitimacy, and identification with responders was entered as: communication condition→identification with responders→perceptions of legitimacy. The model did not fit well with the data, χ^2^(22df) = 61.54, p<.001, suggesting that the initial model, based on the hypothesised relationships, provided a better explanation for the data.

### Qualitative questionnaire data

Results supported the findings from the quantitative questionnaire items, in showing that more participants in the standard practice and brief communication groups reported a need for increased communication and practical information, compared to those in the theory-based communication group (see Table S4).

When asked why they felt nervous or worried during the study, if at all, a far greater proportion of people in the standard practice (38%) and brief (21%) communication conditions reported anxiety due to a lack of communication from emergency responders, compared to those in the theory-based communication condition (0%). The difference between groups was significant *χ^2^*(2df) = 17.81, *p*<.001, *r* = .40. Post-hoc tests revealed that this was due to significantly fewer participants in the theory-based condition reporting feeling nervous due to a lack of communication from responders compared to those in both the standard practice condition (*χ^2^*(1df) = 18.80, p<.001) and the brief condition (*χ^2^*(1df) = 9.83, p<.05).

Nearly a quarter of participants in the brief communication condition (24%) and over a third of participants in the standard practice communication condition (38%) reported that they felt confused, or did not know what they were doing, during the decontamination process. In contrast, only 12% of people in the theory-based communication condition reported feeling confused. The difference between groups was significant *χ^2^*(2df) = 6.68, *p*<.05, *r* = .24. Post-hoc tests revealed that this was due to significantly fewer participants in the theory-based condition reporting feeling confused compared to those in the standard practice condition (*χ^2^*(1df) = 6.72, p<.05). Table S4 shows the number of participants in each group who reported wanting more communication or practical information from responders, feeling confused, or feeling nervous as a result of a lack of communication from responders. The table also shows the percentage of participants in each group who reported each of these aspects.

## Discussion

This study aimed to test the effectiveness of three different responder communication strategies for mass decontamination following a CBRN incident, by using both self-report and behavioural measures. Results support the initial hypotheses in showing that the theory-based communication strategy facilitated the quickest and most efficient progression of volunteers through the decontamination process. This is in line with previous findings in other domains [32], and it is likely to be due to volunteers having a better understanding of what they were required to do during this condition. This is supported by the evidence from the qualitative data analysis and the observational analysis, which revealed that far fewer people in the theory-based communication condition reported feeling confused during the decontamination process, or exhibited confused behaviours, compared to those in the other two conditions. This could have important implications during a real life incident, as confusion could lead to failure to complete the decontamination process successfully, and could result in secondary contamination of other people and places; this could cost lives during a real incident [34], [35].

Many of the hypothesised differences between the theory-based communication condition and the other two conditions were supported, as were predictions about the mediating role of social identity variables between effective responder communication and positive outcome variables (e.g. non-compliance, helping and cooperative behaviour, anxiety). However, there were no significant differences between those in the standard practice and brief communication conditions on any of the variables; possible reasons for this will be discussed in the limitations section below. Results will now be discussed in terms of their implication for each of the outcome variables: compliance; helping and cooperative behaviour; and anxiety.

### Non-compliance

As predicted, results show that effective responder communication resulted in increased levels of compliance, as indicated by both the results from the observational analysis, which showed significantly fewer observations of non-compliant behaviours in the theory-based communication group, and the self-report measures, in which willingness to comply was highest in the theory-based communication condition. However, the increased level of willingness to comply in the theory-based communication condition was not significant. A possible reason for this is that the mean self-reported level of willingness to comply was quite high (*M* = 5.6), significant higher than that recorded in previous similar research [27]. This may have created a ceiling effect, in which it was not possible to determine a significant difference between the three conditions. Possible reasons for the high level of willingness to comply are reported in the limitations section below.

Those in the theory-based communication group reported significantly stronger perceptions of responder legitimacy, and identification with emergency responders, than those in either the standard practice or brief communication groups. Further, results from within-subjects tests revealed that perceptions of responder legitimacy, and identification with emergency responders, increased in the theory-based communication condition, but decreased in the other two conditions, from time 1 to time 2. Perceptions of responder legitimacy, and identification with emergency responders, are factors which have previously been found to be related to levels of compliance [24], [25], and which may have contributed to the reduced number of observations of non-compliant behaviours in the theory-based communication group in the current study. In line with this, path analysis revealed that perceptions of responder legitimacy and identification with emergency responders played a significant mediating role between effective responder communication, perceptions of privacy, and willingness to comply during a real incident. Increasing public compliance with decontamination is of critical importance during real life incidents involving mass decontamination. If members of the public fail to comply with responder instructions, or challenge the authority of emergency responders, this could delay the decontamination process; delayed or inefficient decontamination could result in lives being lost, through prolonged contact with the contaminant, or through secondary contamination of other people and places [34], [35].

### Helping and cooperative behaviour

Results from path analysis support the hypotheses relating to helping and cooperative behaviour, in showing that effective responder communication predicts increased helping and cooperative behaviour, mediated by the social identity variables. Results from between-subjects tests also revealed that self-reported levels of willingness to help others during a real incident were higher in the theory-based communication condition than in the other two conditions, although this was not significant.

However, results from the observational analysis showed increased helping behaviours in the brief communication condition. It is possible that this is because the reduced input from emergency responders in this condition made it more necessary for participants to help each other; in a real incident, there would be far fewer emergency responders to members of the public, so it would likely be more necessary for members of the public to help each other. Further, in the current situation, compliance was very high (participants had all consented to undergo decontamination before the experiment), and therefore ‘helping’ behaviours were directed at helping others to undergo the decontamination process. In a real incident, if identification with other members of the public was high (as in all three groups in the present study), but identification with emergency responders was low (as in the brief communication group), helping among members of the public would still be expected, but this might take a different form. For example, if members of the public do not perceive responders to be behaving legitimately, and therefore do not identify with them, compliance with decontamination is likely to be low. In this case, members of the public might help each other to leave the scene, or challenge the authority of the emergency responders, rather than helping each other to undergo decontamination. It is therefore crucial not only that identification with other members of the public is high (to promote helping and cooperative behaviours), but also that identification with emergency responders is high (to ensure that helping and cooperation among members of the public are directed towards undergoing decontamination).

Cooperative and helping behaviour among members of the public is likely to be important in order to facilitate the smooth-running of the decontamination process during a real incident involving mass decontamination; in an incident of this type, emergency responders will have limited time and resources, and it will therefore be crucial that members of the public cooperate with and help each other when necessary, in order to successfully undergo decontamination.

### Anxiety

Results from the qualitative questionnaire measures reveal that nearly a quarter of participants in the brief communication condition, and over a third of participants in the standard practice communication condition, reported that they felt anxious due to a lack of communication from responders; in contrast, no volunteers reported that they felt anxious due to a lack of communication from responders in the theory-based communication condition. This is in line with results from small scale incidents involving decontamination, in showing that a lack of communication from responders contributed to increased anxiety [5], [6]. Results of within-subjects tests support this, in showing that those in the theory-based communication condition reported a significant reduction in expectations of anxiety during a real incident, from time 1 to time 2.

However, results from path analysis failed to show support for the predicted relationships between the social identity variables and anxiety. Measures were taken of both actual anxiety experienced during the process, and expected anxiety during a real incident. As in previous research [21], anxiety reported on the quantitative self-report measure was very low (*M* = 3.24), significantly below the midpoint value of 4, creating difficulty in establishing any relationships between actual anxiety and the other variables. In contrast, the mean for expectations of anxiety during a real incident was very high (5.50), significantly higher than actual anxiety experienced, and significantly higher than the scale midpoint. The fact that expectations of anxiety were so high, and that there was a large difference between actual anxiety and expected anxiety, suggests that it may be difficult for members of the public to accurately imagine how anxious they would feel during an incident of this type; there may be a tendency to automatically assume a very high level of anxiety. This may therefore explain why the expected relationships between anxiety and the relevant variables were not present in the self-reported quantitative measures.

### Implications

#### Theoretical implications

Decontamination has traditionally been seen as a technical issue [9], with very little effort to understand how members of the public are likely to behave during such incidents. Where psychosocial issues have been considered, policy makers and planners have tended to rely on assumptions of ‘mass panic’; this has led to a focus on controlling, rather than communicating with, members of the public [9]. Planning for incidents involving mass decontamination has therefore lacked an understanding of the likely psychosocial issues involved, and has been based on outdated assumptions, rather than evidence. By applying the social identity approach during a simulated incident involving mass decontamination, the current research has been able to test the effectiveness of three different responder communication strategies, and to show how and why the provision of health-focused communication, and practical information, is so important during incidents involving mass decontamination.

The findings from this research provide support for the social identity model of collective resilience [18], [20], by showing a significant relationship between identification with other members of the public, collective agency, and increased willingness to help others. The results also provide support for the elaborated social identity model [23], in showing that effective responder communication results in increased perceptions of responder legitimacy, which in turn increases identification with emergency responders. This research therefore shows that aspects of the social identity model of collective resilience (SIMCR) and the ESIM are applicable during incidents involving mass decontamination, and that these two theories can be combined to create a model of likely crowd behaviour during incidents involving mass decontamination.

#### Practical implications

The results show that a communication strategy which includes honest information about the actions emergency responders are taking, health-focused information about decontamination, and sufficient practical information, results in improved outcomes in terms of both the perceptions, and the behaviour, of members of the public. The results suggest that such a communication strategy will result in increased speed and efficiency of the decontamination process, increased compliance, reduced anxiety, and increased cooperative behaviour among members of the public. These factors are likely to save lives during a real incident involving decontamination, as any delay in the decontamination process will result in the increased potential for adverse health effects from the contaminant. When managing an incident involving mass decontamination, emergency responders should therefore strive to: communicate openly and honestly with members of the public; provide health-focused information about decontamination, including about the benefits of decontamination; and provide sufficient practical information during the decontamination process.

### Limitations

There are several potential limitations of this research. First, this was a simulated incident, in which participants knew that no harm was going to come to them. This may have had an important impact on certain variables, such as anxiety and compliance. In particular, it is likely that anxiety would be higher during a real incident, and it is possible that this may affect the way in which members of the public behave during a real incident, and may affect relationships between other variables. However, as noted in the introduction, while anxiety may be increased during real life emergencies, there is very little evidence that members of the public are likely to panic during mass emergencies of this type. Further, available evidence suggests that anxiety can be reduced by the provision of sufficient information from emergency responders. Thus, while the low level of anxiety during the present study made it difficult to examine relationships between anxiety and the other variables, there is no evidence that increased anxiety during a real incident would change the nature of the relationships between the other variables; indeed, effective responder communication is likely to be more important, rather than less, if anxiety is higher.

Second, it may be questioned whether the groups in which people underwent the decontamination process (n≤42) during this study were of a sufficient size as to represent ‘mass’ decontamination. However, it has been suggested that incidents of up to 50 victims may be defined as ‘small-scale mass casualty incidents’, indicating that the group sizes used during the current study were of sufficient size as to be termed ‘mass’ [36]. Further, it is likely that the importance of effective responder communication strategies, and the mediating role played by social identity variables, would have been more evident, rather than less, had the groups been larger.

Third, the differences between the standard practice and brief communication conditions were not obvious enough to participants. While the theory-based condition was perceived as being significantly more effective than the other two conditions, there were no significant differences in perceptions of the standard practice and brief conditions. In the brief condition and the theory-based condition responders understood that they were either to provide no extra information (brief condition) or any extra information they felt was required (theory-based condition). However, responders were not clear about how much they were allowed to say to participants in the standard practice communication group, and as a result gave less information than expected, thus resulting in less difference between the brief and standard practice conditions than intended. It would therefore be beneficial to run a future study, in which emergency responders receive a more precise brief about the way in which they should communicate with participants in the standard practice condition. This would enable conclusions to be drawn about the effectiveness of providing practical information alone, compared to providing both practical information and increased communication.

A fourth possible limitation is that participants received information about decontamination (including why and how decontamination would be carried out), prior to taking part in the study. This may therefore have resulted in a greater willingness to comply, due to a greater understanding of the need for the process. This is supported by the fact that the mean level of compliance in this study was quite high. Future research should therefore provide less information to participants prior to the study about the importance of decontamination, whilst ensuring that participants still have enough information about the nature of the study to be able to give their informed consent. Relatedly, participants' level of compliance may also have been affected by the fact that participants were only required to strip to their swimwear; in a real incident, they would be asked to undergo the process naked. Even stripping to swimwear in public is embarrassing for members of the public [21]; being asked to strip naked would be even more embarrassing, and it is likely that compliance would be lower during a real incident as a result of this. However, we argue that the results of the current study are even more important in light of this. If a perception of responder legitimacy (based on effective responder communication and the provision of sufficient privacy) affects increased willingness to comply during a simulated incident, in which participants were required to strip only to their swimwear, and knew no real harm was going to come to them, it is likely to be even more important during a real incident.

A final limitation to note is that, while the observational analysis was carried out by two independent researchers, who were not members of the study research team, the observers were not blind to which condition was which. It is therefore possible that researchers' observations were biased due to this. However, observers were unaware of the aims of the study, making bias less likely.

## Conclusion

Overall, this study shows that communication strategies which are perceived by members of the public as the most effective are those which include health-focused explanations about the decontamination process, information about the actions responders are taking, and sufficient practical information. A communication strategy which encompasses these aspects is likely to increase the speed and efficiency of the decontamination process, by improving both public willingness, and ability, to take recommended actions. Increasing the speed and efficiency of the decontamination process may result in lives being saved during a real life incident of this type.

## Supporting Information

Table S1 **Time taken (in minutes) for each group to progress through the decontamination process in the three different communication conditions.**
(DOC)

Table S2 **Observed behaviours within each of the three different communication conditions.**
(DOC)

Table S3 **Mean scores of all measures at time 2 for the three different communication conditions.**
(DOC)

Table S4 **Results of qualitative questionnaire data from each of the three different communication conditions.**
(DOC)

Appendix S1 **Scenario for participants.**
(DOC)

Appendix S2 **Communication messages.**
(DOC)

Appendix S3 **Content analysis qualitative questionnaire data.**
(DOC)

Appendix S4 **Content analysis observational data.**
(DOC)

Appendix S5 **Table of timing data for each small group within each of the three conditions.**
(DOC)
