# Supplementary material for: Effective Responder Communication Improves Efficiency and Psychological Outcomes in a Mass Decontamination Field Experiment: Implications for Public Behaviour in the Event of a Chemical Incident
Source: PLoS One. 2014 Mar 4;9(3):e89846. doi: 10.1371/journal.pone.0089846 (PMC3942378; doi:10.1371/journal.pone.0089846)
Supplement: Table S1 — Time taken (in minutes) for each group to progress through the decontamination process in the three different communication conditions. (DOC) [file pone.0089846.s001.doc]

| **Condition** | **M** | **SD** | **Min** | **Max** | **Range** |
| --- | --- | --- | --- | --- | --- |
| Theory-based communication | 10.80 | .67 | 10.00 | 11.50 | 1.50 |
| Standard practice communication | 14.84 | 3.42 | 11.80 | 18.30 | 6.50 |
| Brief communication | 11.83 | 5.34 | 8.50 | 19.80 | 11.30 |
