# Supplementary material for: Effective Responder Communication Improves Efficiency and Psychological Outcomes in a Mass Decontamination Field Experiment: Implications for Public Behaviour in the Event of a Chemical Incident
Source: PLoS One. 2014 Mar 4;9(3):e89846. doi: 10.1371/journal.pone.0089846 (PMC3942378; doi:10.1371/journal.pone.0089846)
Supplement: Table S2 — Observed behaviours within each of the three different communication conditions. (DOC) [file pone.0089846.s002.doc]

| **Condition** | **Non-compliance** | **Confusion** | **Helping** |
| --- | --- | --- | --- |
| Theory-based communication | 2 | 31 | 8 |
| Standard practice communication | 5 | 44 | 10 |
| Brief communication | 21 | 66 | 16 |
