# Supplementary material for: Effective Responder Communication Improves Efficiency and Psychological Outcomes in a Mass Decontamination Field Experiment: Implications for Public Behaviour in the Event of a Chemical Incident
Source: PLoS One. 2014 Mar 4;9(3):e89846. doi: 10.1371/journal.pone.0089846 (PMC3942378; doi:10.1371/journal.pone.0089846)
Supplement: Table S3 — Mean scores of all measures at time 2 for the three different communication conditions. (DOC) [file pone.0089846.s003.doc]

| **Variable** | **Brief communication** | | **Standard practice communication** | | **Theory-based communication** | |
| --- | --- | --- | --- | --- | --- | --- |
|  | **M** | **SD** | **M** | **SD** | **M** | **SD** |
| **Legitimacy** | 4.85 | 1.50 | 5.01 | 1.28 | 6.41 | .67 |
| **Identification responders** | 2.83 | 1.16 | 3.17 | 1.33 | 4.12 | 1.37 |
| **Identification public** | 5.27 | .86 | 5.10 | 1.21 | 4.98 | 1.21 |
| **Collective agency** | 5.77 | .82 | 5.88 | 1.31 | 6.24 | 1.01 |
| **Compliance** | 5.45 | 1.28 | 5.61 | 1.23 | 5.68 | 1.30 |
| **Actual anxiety** | 3.31 | 1.29 | 3.15 | 1.43 | 3.17 | 1.32 |
| **Expectations anxiety** | 5.73 | .91 | 5.72 | 1.26 | 5.11 | 1.46 |
| **Willingness to help others** | 6.09 | .95 | 6.34 | 1.00 | 6.37 | .77 |
| **Privacy** | 4.62 | 1.59 | 4.91 | 1.71 | 4.31 | 1.83 |
