# Supplementary material for: Effective Responder Communication Improves Efficiency and Psychological Outcomes in a Mass Decontamination Field Experiment: Implications for Public Behaviour in the Event of a Chemical Incident
Source: PLoS One. 2014 Mar 4;9(3):e89846. doi: 10.1371/journal.pone.0089846 (PMC3942378; doi:10.1371/journal.pone.0089846)
Supplement: Table S4 — Results of qualitative questionnaire data from each of the three different communication conditions. (DOC) [file pone.0089846.s004.doc]

| Condition | Wanted more communication (%) | Wanted more practical info (%) | Anxiety – no communication (%) | Confusion (%) |
| --- | --- | --- | --- | --- |
| Theory-based communication | 5 (12) | 15 (36) | 0 (0) | 5 (12) |
| Standard practice communication | 24 (75) | 26 (81) | 12 (38) | 12 (38) |
| Brief communication | 24 (63) | 26 (68) | 8 (21) | 9 (24) |
