# Supplementary material for: Effective Responder Communication Improves Efficiency and Psychological Outcomes in a Mass Decontamination Field Experiment: Implications for Public Behaviour in the Event of a Chemical Incident
Source: PLoS One. 2014 Mar 4;9(3):e89846. doi: 10.1371/journal.pone.0089846 (PMC3942378; doi:10.1371/journal.pone.0089846)
Supplement: Appendix S1 — Scenario for participants. (DOC) [file pone.0089846.s005.doc]

**Appendix 1: Scenario for participants**

Imagine you are waiting in a crowded lecture theatre for a lecture to begin. Whilst you are waiting, an announcement plays over the loudspeakers in the lecture theatre. You are told that a suspicious package has been delivered to the building. You are told that the package contains a suspicious substance, and you are asked to remain in the building until further notice. After some time, emergency responders wearing protective equipment enter the lecture theatre. They direct you and the others in the lecture theatre to move outside.

As you leave the building you hear sirens, and the Fire and Rescue Service arrive at the scene. You notice that all the emergency responders are wearing hazard protection suits. Fire and Rescue Service crew members move everyone into an area a little way away from the lecture building, and ask people not to leave the scene. Other members of the Fire and Rescue Service are setting up a decontamination shower a short way away, and are directing people towards it. As you are moved closer to the decontamination tent, you see others who were involved in the incident undressing as they are about to go through the decontamination shower. You realise that you will be expected to remove your clothes before going through the shower. A crowd has formed around the decontamination shower, and onlookers are watching the decontamination process take place.
