# Supplementary material for: Effective Responder Communication Improves Efficiency and Psychological Outcomes in a Mass Decontamination Field Experiment: Implications for Public Behaviour in the Event of a Chemical Incident
Source: PLoS One. 2014 Mar 4;9(3):e89846. doi: 10.1371/journal.pone.0089846 (PMC3942378; doi:10.1371/journal.pone.0089846)
Supplement: Appendix S2 — Communication messages. (DOC) [file pone.0089846.s006.doc]

**Appendix 2: Communication messages**

*Theory-based communication message*

*At the start*:

As a precaution, we would like you to undergo a decontamination shower. This is important, as it will remove any contaminant which may be present on your skin. Before going through the decontamination shower, we would like you to remove the outer layers of your clothes, down to your swimwear. By removing your outer layers of clothing, you will be removing up to 90% of any contaminant which you may have come into contact with. Undergoing a decontamination shower will then remove any remaining contaminant from your skin. This will help to prevent you suffering any adverse effects from the contaminant.

Please take off your outer layers of clothes, down to your swimwear. Please then open the packs which are on the ground in front of you. Please change into the orange ponchos, and place the clothes you have taken off into the bag provided. Further instructions are provided within the packs.

*After 5 minutes:*

The decontamination process will begin as soon as possible. Please be patient while we finish setting up the decontamination tent. Thank you for your co-operation.

*After 10 minutes:*

The decontamination process will begin as soon as possible. During this time, please make sure you have changed into the orange poncho provided, and have placed the clothes you were wearing into the bag provided. This will remove 90% of any contaminant which you may have come into contact with. Thank you for your co-operation.

*After 15 minutes:*

We are now finishing the set up of the decontamination tent, and the decontamination process will begin in about 5 minutes time. It is important that you undergo a decontamination shower, as this will remove any contaminant which may remain on your skin. We appreciate your patience, and the decontamination process will begin as soon as possible. Thank you for your co-operation.

*After 20 minutes:*

We have now finished setting up the decontamination tent, and the decontamination showering process will now begin. Thank you for your patience.

*During the decontamination process (repeat when each group is waiting to go through the decontamination process):*

When the light on the outside of the decontamination shower turns green, and an audible signal is heard, you should enter the first section of the decontamination shower in groups of five. You should then remove the orange poncho you are wearing, and place it on the floor. When the light at the entrance to the next section of the tent turns green, move forward into the showering section of the decontamination tent. You will have three minutes to wash yourself in the showering section of the decontamination tent, and you should wash from head to toe. When the red light at the end of the showering section turns green, you should leave the showering section and move into the final section of the decontamination unit. Within this section you will find some clothing packs containing clothing for you to get dressed into. When the red light changes to green, you should leave the decontamination tent. Responders will meet you on the other side of the decontamination unit, and will explain what you should do next.

*Standard practice communication message*

*At the start:*

We would like you to undergo a decontamination shower. Please take off your outer layers of clothes, down to your swimwear. Please then open the packs which are on the ground in front of you. Please change into the orange ponchos, and place the clothes you have taken off into the bag provided. Further instructions are provided within the packs.

*After 20 minutes:*

We have now finished setting up the decontamination tent, and the decontamination showering process will now begin.

*During the decontamination process (repeat when each group is waiting to go through the decontamination process):*

When the light on the outside of the decontamination shower turns green, and an audible signal is heard, you should enter the first section of the decontamination shower in groups of five. You should then remove the orange poncho you are wearing, and place it on the floor. When the light at the entrance to the next section of the tent turns green, move forward into the showering section of the decontamination tent. You will have three minutes to wash yourself in the showering section of the decontamination tent, and you should wash from head to toe. When the red light at the end of the showering section turns green, you should leave the showering section and move into the final section of the decontamination unit. Within this section you will find some clothing packs containing clothing for you to get dressed into. When dressed you should leave the decontamination unit. Responders will meet you on the other side of the decontamination unit, and will explain what you should do next.

*Brief communication message*

*At the start:*

We would like you to undergo a decontamination shower. Please take off your outer layers of clothes, down to your swimwear. Please then change into the orange ponchos provided.

*After 20 minutes:*

We have now finished setting up the decontamination tent, and the decontamination showering process will now begin.

*During the decontamination process (repeat when each group is waiting to go through the decontamination process):*

When the light outside the decontamination tent changes, you should enter the first section of the decontamination unit in groups of five, and remove the clothes you are wearing. Once you have removed your clothes, you should enter the decontamination shower. Wash yourself. Following this, you should leave the showering unit and re-dress in the clothes provided.
