# Supplementary material for: Effective Responder Communication Improves Efficiency and Psychological Outcomes in a Mass Decontamination Field Experiment: Implications for Public Behaviour in the Event of a Chemical Incident
Source: PLoS One. 2014 Mar 4;9(3):e89846. doi: 10.1371/journal.pone.0089846 (PMC3942378; doi:10.1371/journal.pone.0089846)
Supplement: Appendix S3 — Content analysis qualitative questionnaire data. (DOC) [file pone.0089846.s007.doc]

**Appendix 3: Content analysis qualitative questionnaire data**

| **Wanting more communication** | **Quotes** |
| --- | --- |
| Any quotes in which participants specifically stated that they would have liked more communication from emergency responders | *“Someone could have told us why we were waiting and for how long we were to wait.”*  *“[There was] no explanation of why the showers will ‘help’ – what would have been in the water?”*  *“A lot more clarity of actions needed. More reassurance. More communication as to what was going on.”* |
| **Wanting more practical information** |  |
| Any quotes in which participants specifically stated that they would have liked more practical information to be provided during the decontamination process | *“I was unsure exactly how to dress in the beginning, verbal instructions would be helpful.”*  *“It is a new experience, so having spoken directions about what is needed would have been useful and also put us at ease.”*  *“It is advisable to have emergency responders showing how to use each item before entering the shower.”* |
| **Feeling confused** |  |
| Any quotes in which participants specifically stated that they felt confused or did not know what they were supposed to do at some point during the decontamination process | *“I was confused as to what I was supposed to do after the shower – there were no instruction sheets.”*  *“[Need] people talking to us and telling us what to do – didn’t know what we were doing most of the time.”*  *“I did several things incorrectly because I was confused. It would be better if someone explained to us what to do.”* |
| **Feeling anxious as a result of a lack of communication from responders** |  |
| Any quotes in response to the question “If you felt nervous or worried, please describe what the main reason for this was” which indicated that the main reason for participants’ feeling nervous was a lack of communication from responders | *“No communication between us and emergency services. I wasn’t sure they knew what they were doing.”*  *“The information given was obviously not the whole story and gaps in information make people worried.”*  *“Uncertainty – no information given explaining how long we were going to be standing around for.”* |
