# Supplementary material for: Effective Responder Communication Improves Efficiency and Psychological Outcomes in a Mass Decontamination Field Experiment: Implications for Public Behaviour in the Event of a Chemical Incident
Source: PLoS One. 2014 Mar 4;9(3):e89846. doi: 10.1371/journal.pone.0089846 (PMC3942378; doi:10.1371/journal.pone.0089846)
Supplement: Appendix S4 — Content analysis observational data. (DOC) [file pone.0089846.s008.doc]

**Appendix 4: Content analysis observational data**

| **Non-compliant behaviours** |
| --- |
| Participants refusing to carry out instructions (e.g. refusing to leave the rerobe section of the decontamination shower when asked to do so, refusing to wear the face masks provided, refusing to take glasses off etc). |
| Participants leaving the immediate trial area during disrobe, although asked not to do so by responders |
| Participants re-entering the rerobe section of the decontamination shower, although asked not to do so by responders. |
| **Helping behaviours** |
| Participants helping others to open disrobe and rerobe packs |
| Participants helping others to get changed |
| Participants helping others to read the instructions provided |
| **Confusion** |
| Participants looking to see what others are doing before carrying out any actions |
| Participants asking other participants to explain what they should be doing |
| Participants asking emergency responders to explain what they should be doing |
