# Supplementary material for: Effective Responder Communication Improves Efficiency and Psychological Outcomes in a Mass Decontamination Field Experiment: Implications for Public Behaviour in the Event of a Chemical Incident
Source: PLoS One. 2014 Mar 4;9(3):e89846. doi: 10.1371/journal.pone.0089846 (PMC3942378; doi:10.1371/journal.pone.0089846)
Supplement: Appendix S5 — Table of timing data for each small group within each of the three conditions. (DOC) [file pone.0089846.s009.doc]

**Appendix 5: Table of timing data for each small group within each of the three conditions**

| **Condition** | **Group** | **Time (minutes)** |
| --- | --- | --- |
| **Brief** | 1 | 9.5 |
|  | 2 | 8.5 |
|  | 3 | 9.5 |
|  | 4 | 19.75 |
| **Standard practice** | 1 | 11.75 |
|  | 2 | 12 |
|  | 3 | 17.25 |
|  | 4 | 18.34 |
| **Theory-based** | 1 | 10 |
|  | 2 | 10.5 |
|  | 3 | 10.5 |
|  | 4 | 11.5 |
|  | 5 | 11.5 |
